# Land use for animal production in global change studies: Defining and characterizing a framework

**DOI:** 10.1111/gcb.13732

**Published:** 2017-06-01

**Authors:** Leanne N. Phelps, Jed O. Kaplan

**Affiliations:** ^1^ Institute of Earth Surface Dynamics University of Lausanne Lausanne Switzerland

**Keywords:** animal production, browsing, global change studies, grazing, land cover, land use, livestock, modeling, pasture, rangeland

## Abstract

Land use for animal production influences the earth system in a variety of ways, including local‐scale modification to biodiversity, soils, and nutrient cycling; regional changes in albedo and hydrology; and global‐scale changes in greenhouse gas and aerosol concentrations. Pasture is furthermore the single most extensive form of land cover, currently comprising about 22–26% of the earth's ice‐free land surface. Despite the importance and variable expressions of animal production, distinctions among different systems are effectively absent from studies of land use and land cover change. This deficiency is improving; however, livestock production system classifications are rarely applied in this context, and the most popular global land cover inventories still present only a single, usually poorly defined category of “pasture” or “rangeland” with no characterization of land use. There is a marked lack of bottom‐up, evidence‐based methodology, creating a pressing need to incorporate cross‐disciplinary evidence of past and present animal production systems into global change studies. Here, we present a framework, modified from existing livestock production systems, that is rooted in sociocultural, socioeconomic, and ecological contexts. The framework defines and characterizes the range of land usage pertaining to animal production, and is suitable for application in land use inventories and scenarios, land cover modeling, and studies on sustainable land use in the past, present, and future.

## 1 INTRODUCTION

It is widely acknowledged that land use is a major driver of environmental change at local, regional, and global scales, with important impacts on biogeochemical cycling, ecosystem structure and function, and greenhouse gas emissions (Foley et al., 2005; Herrero et al., 2016; Rockström, Gordon, & Folke, 1999; Sala et al., 2000; Steinfeld et al., 2006; Vitousek, Mooney, Lubchenco, & Melillo, 1997). Land dedicated to animal production is crucial for supporting dietary needs worldwide, contributing at least 40% of the global agricultural output and securing livelihoods for nearly 1.3 billion people (Steinfeld et al., 2006). Land use/cover datasets have identified pasture or rangeland as the most extensive form of used land, accounting for about 22–26% of the earth's ice‐free land surface (e.g., Ellis, Klein Goldewijk, Siebert, Lightman, & Ramankutty, 2010; Ellis & Ramankutty, 2008; Klein Goldewijk, Beusen, & Janssen, 2010; Klein Goldewijk & Ramankutty, 2004; Ramankutty, Evan, Monfreda, & Foley, 2008), and having enormous influences on terrestrial ecosystems (Asner, Elmore, Olander, Martin, & Harris, 2004; Erb et al., 2007; Vitousek, Ehrlich, Ehrlich, & Matson, 1986). Despite the importance of animal production, the magnitude, distribution, and history of its influence on the earth system are poorly understood. This lack of understanding partly arises from the absence of clear definitions for animal production practices, and the influence these have on landscapes in the past and present. The terms “pasture” and “rangeland” are commonly used in global change studies to indicate areas that have been modified by animal production, namely grazing (e.g., Lambin et al., 2001); however, both terms take on a variety of definitions and interpretations (Table S1), which produce varied results between studies.

The other main limitation to our understanding of the impact of animal production on land cover arises from the difficulty in detecting land use in space and understanding how land use changes over time. Here we refer to two primary types of datasets that characterize the human influence on the earth's land surface: Land Use/Land Cover (LULC) and Anthropogenic Land Cover Change (ALCC). LULC datasets provide information for a given time, and are derived from a variety of primary data sources. For example, the LULC datasets GLC2000 (Bartholomé, 2005) and GlobCover2009 (Bontemps et al., 2011) are based on remote sensing, FAOSTAT is based on statistical inventory data (see FAO, 2015), and the Ramankutty et al. (2008) dataset relies on a combination of remote sensing and statistical inventory data. Alternatively, ALCC datasets represent land use *change* over time, for example, HYDE 3.1 (Klein Goldewijk et al., 2010) or KK10 (Kaplan et al., 2010). While GLC2000 does inventory some types of land managed for animal production by combining expert knowledge with remote sensing at a regional level, all of the datasets listed above are affected by problems of definition and characterization and by difficulties in detecting land use remotely, particularly for animal production. As a result, land used for animal production may be falsely classified as “seminatural” or “natural” (e.g., Klein Goldewijk, 2001; Lambin, Geist, & Lepers, 2003; Pongratz, Reick, Raddatz, & Claussen, 2008), and no ALCC or LULC dataset accurately or explicitly represents different forms of animal production.

Perhaps the most fundamental problem with both ALCC and LULC datasets is that land cover and use are typically conflated (Erb et al., 2007, 2013; Verburg, Neumann, & Nol, 2011; Verburg, Van De Steeg, Veldkamp, & Willemen, 2009). In some cases, land use leaves an unambiguous impact on land cover, for example, conversion of natural forest land to arable fields. In many other cases, however, it is extremely difficult to associate a particular land cover with its land use; this is especially problematic when trying to associate animal production with a particular land cover. Thus, ground‐truthing global pasture and rangeland datasets is plagued by imprecise definitions, varied classification systems, scaling problems, and temporal and spatial inconsistencies associated with difficulty in detecting land use change over time (Verburg et al., 2011). Even where datasets have been ground‐truthed, “open land cover” is prone to significant inaccuracies in terms of classification (Bach et al., 2006). These uncertainties, limitations, and biases must be explicitly stated and critically examined in order to avoid misuse in practical application, for example, in setting policies (e.g., Bach et al., 2006; Dendoncker, Schmit, & Rounsevell, 2008; Fassnacht, Cohen, & Spies, 2006; Petz et al., 2014).

In section two of this paper, we specify the problems associated with variable definitions of land use for animal production, and the lack of animal production characterization in studies of land use and cover change. In section three, we discuss the uncertainties and biases associated with remote sensing (RS) and statistical inventory (SI) data for modern times, and in section four, we outline the uncertainties and biases of pasture estimations for the preindustrial past. In section five, we present a comprehensive, cross‐disciplinary framework for more accurately considering and incorporating animal production systems into land use inventories for both the present and the past.

## 2 PROBLEMS WITH DEFINITIONS AND CHARACTERIZATIONS

Global estimates of pasture and rangeland extent are highly variable due to the use of imprecise definitions in global change studies (Table S1). Most definitions do not explicitly disentangle land use from cover (e.g., Allen et al., 2011). For example, the widely used Food and Agriculture Organization of the United Nations (FAO) definition of pasture is “…the land used permanently (for a period of five years or more) for herbaceous forage crops, either cultivated or naturally growing” (FAOSTAT, 2014). When this definition of permanent pasture is applied, the distinction between land cover types is indefinite, resulting in varying inclusions and exclusions between datasets and statistical offices. Furthermore, browse land, that is, shrubs, trees, and succulents that are consumed for animal production, is typically excluded from this grazing‐focused definition. While it is possible to consider grazed land separately from browsed land, it is not possible to separate grazing and browsing land use, as most animal production systems include a combination of the two.

In order to properly quantify the extent and intensity of regional and global environmental modifications from animal production, land use systems must be accurately characterized. Animal production and cultivation are often treated as mutually exclusive categories for ease of use, that is, cropland vs. pasture (Asselen & Verburg, 2012; Letourneau, Verburg, & Stehfest, 2012; Monfreda, Ramankutty, & Foley, 2008), even though this does not reflect the reality of most land use systems, and even though a number of production system classifications exist (e.g., Asselen & Verburg, 2012; Letourneau et al., 2012; Monfreda et al., 2008; Otte & Chilonda, 2002; Robinson et al., 2011; Seré, Steinfeld, & Groenewold, 1996). While many classifications incorporate mixed crop–livestock systems, they may not consider all details of land use intensity, and are likely to combine socioeconomic and environmental variables. In reality, social and environmental factors are often inextricably linked; however, in order to investigate the complex interplay between land use and cover change, it is important to consider them separately—especially when dealing with change over long periods of time, where it is necessary to reconstruct both environmental and social attributes using proxies.

## 3 PROBLEMS WITH DATASETS REPRESENTING CONTEMPORARY LAND USE

Although attention has been given to some of the technical problems associated with LULC and ALCC datasets (Verburg et al., 2011), land use for animal production requires further consideration. The effects of animal production on land cover are underrepresented in studies of land use and land cover change. For example, the primary type of land cover associated with animal production is typically referred to as a homogenous category of pasture or rangeland, but animal production is an important part of many other biomes, such as savannas, deserts, forests, and even tundra. The influence of animal production systems on the landscape is furthermore non‐negligible; pastoralism is often associated with anthropogenic manipulations that affect land cover both directly, for example, digging wells, fire management, and draining wetlands, and indirectly, for example, through herd management strategies (Borger, 1992; Dahl & Hjort, 1976; Homewood, 2008). Where LULC datasets do identify pasture or rangeland (e.g., Ellis et al., 2010), the wide variety of animal production strategies practiced on these landscapes is usually not acknowledged. Below, we outline the limitations of RS and SI data in representing the various forms of land use for animal production.

### 3.1 Datasets based on remote sensing

While a variety of limitations prevent RS data from accurately representing the extent and intensity of animal production, it must first be clear that RS‐based land cover categories do not inherently represent *land use,* even though they are frequently treated as such. Instead, RS categories represent mutually exclusive types of *land cover*, which contain limited information about the type and intensity of land use, especially in the case of animal production (Bach et al., 2006; Brown & Duh, 2004; Erb et al., 2007; Verburg et al., 2011). When land use is not explicitly considered, a variety of interpretations, inclusions, and exclusions are made between datasets (Table S1, supporting discussion on Fig. S1), leading to varied results. This is exemplified in that a large portion of the land cover mapped in RS‐based datasets, especially mixed and open land use/cover categories, are poorly discriminated between datasets in terms of spatial agreement and class accuracy (Bach et al., 2006; Herold, Mayaux, Woodcock, Baccini, & Schmullius, 2008; Verburg et al., 2011). Most RS‐based global land cover datasets have low to medium spatial resolution, which is likely to show industrial‐scale land cover changes – such as homogenous land cover generated from intensive ranching – but underrepresents those associated with small‐scale land use (Dendoncker et al., 2008; Ellis et al., 2009; Fassnacht et al., 2006; Hurtt et al., 2006; Ozdogan & Woodcock, 2006), such as land cover mosaics generated from pastoralism and stock‐keeping (Lambin, 1999). While locally difficult to detect, these small‐scale changes, taken as a whole, have large implications for global land cover. This limitation can be mitigated in the future with higher resolution RS technology (e.g., Hansen et al., 2013). Still, RS can neither distinguish between many types of vegetation cover nor explicitly account for land use intensity, for example, by sensing stocking density or the species present (e.g., Fritz et al., 2011; Ramankutty & Foley, 1998). RS data are also generally incapable of detecting land use beneath a forest canopy, resulting in the exclusion of significant areas of land that may be used by domesticated browsers from LULC maps. This leads to a general underestimation of the impact of browsers on vegetation (see Adams, 1975).

### 3.2 Datasets based on statistical inventory

SI data are often used to distribute anthropogenic land use spatially, because it provides relatively low‐cost quantitative estimates on a continuous global scale; nevertheless, SI data are error‐prone for a number of reasons (Erb et al., 2007; Hurtt, Rosentrater, Frolking, & Moore, 2001). Variable definitions and interpretations of land use lead to variable SI datasets, for example, between country‐level, subnational, and FAO statistics. Furthermore, large ecologically and socioculturally irrelevant administrative units are often used, although this may be improved somewhat using administrative subunits (e.g., Ellis & Ramankutty, 2008). SI is also plagued by certain data quality issues, especially for developing countries or where subsistence information is neglected. For example, SI data quality in Africa has been poor and/or inconsistent since the 1960s for a number of reasons (Randall, 2015): first, mobility is a key characteristic of many pastoralist livelihoods, which makes it difficult to find and count people and their animals, and has led to large‐scale exclusions from national censuses (Randall, 2008). Second, mobile people are liable to move across national boundaries, or may be associated with war zones where demographic data are unlikely to be collected. For example, insurgencies and civil wars in Ethiopia resulted in the omission of pastoral zones from the 1984 census data (Central Statistical Authority Ethiopia, 1984 in Randall, 2015). Third, modern pastoralists are often marginalized and may be purposefully excluded from censuses or actively avoid demographic representation altogether (Randall, 2015). Finally, published statistics may even be deliberately manipulated, for example, to suppress subpopulation numbers, although these types of exclusions have been reduced in recent years. Little information has been published on SI data gaps for mobile populations outside of Africa; however, underrepresentation or exclusion of mobile pastoralist societies is likely in countries where mobile pastoralism is not a well‐represented livelihood (Randall, 1993, 2008, 2015).

**Figure 1:**
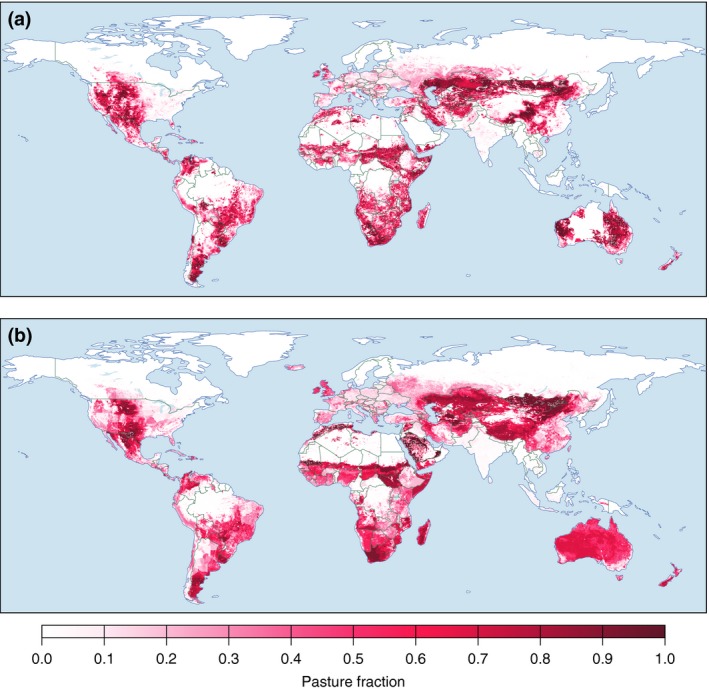
Pasture fraction in two widely used global land use datasets for the year 2000: (a) R2000 (Ramankutty et al., 2008); (b) HYDE 3.1 (Klein Goldewijk et al., 2010) [Colour figure can be viewed at wileyonlinelibrary.com]

While SI and RS data are compatible with each other, neither reflects the complete reality of animal production. As discussed, data quality, definition, and characterization issues associated with both types of data yield highly mixed and uncertain results for estimation of animal production extent and intensity. We illustrate this by comparing two widely used LULC datasets for the year 2000 (HYDE 3.1: Klein Goldewijk et al., 2010; and R2000: Ramankutty et al., 2008) in terms of pasture fraction (Figure 1a, b) and pasture‐associated land cover type (Fig. S1): see Appendix S1 for further discussion.

## 4 PROBLEMS WITH DATASETS REPRESENTING PREHISTORIC AND HISTORIC LAND USE

Accounting for land use is more complicated when considering animal production in the historic and prehistoric past, where RS and SI data are largely unavailable. The same problems exist with defining and characterizing animal production systems as in the contemporary world; however, there are more uncertainties in the past. These include unfamiliar constraints, intensity, and expressions of land use, for which a complete accounting requires numerous lines of evidence.

Animal production has many different expressions of land use, that is, varying production strategies and intensity, but ALCC datasets infer these changes with simple underlying assumptions, which do not accurately represent the complicated temporal dynamics of land use. These limitations may be improved somewhat in contemporary times by aggregating RS observations over several seasons or multiple years; however, these data are generally unavailable for the past. Several global ALCC datasets exist that cover part or all of the preindustrial Holocene, but none of these explicitly consider the variety of animal production systems that existed in the past (Ellis & Ramankutty, 2008; Ellis et al., 2010; Kaplan et al., 2010; Klein Goldewijk et al., 2010; Lemmen, 2009; Mann, Dana, & Doolittle, 2013; Olofsson & Hickler, 2007; Pongratz et al., 2008). Those datasets that contain maps of “pasture” categorize this land use in the same homogenous way as they do for the present, and show very large regional disagreement between studies. The most widely used historical ALCC dataset, HYDE 3.1 (Klein Goldewijk et al., 2010), contains maps of “pasture,” but does not accurately reflect the presence of historic and prehistoric animal production. For example, it estimates very little pasture in the Sahara before 3000 BC, although archeological evidence shows that animal production was already widespread millennia before this time (e.g., Kuper & Kropelin, 2006). Furthermore, the historical ALCC datasets cited above treat land use as a scalar variable ranging from 0 to 100%, and do not acknowledge animal production systems or their varying effects on different environments.

While there is an obvious need for the incorporation of land use evidence, for example, in archeological and paleoecological archives, this process is not straightforward as evidence of mobile rangeland use can be elusive (Chang & Koster, 1986; Fauvelle‐Aymar, Sadr, Bon, & Gronenborn, 2006; Macdonald, 1999; Smith, 2005). For example, pastoralism can be difficult to detect in archeological records due to sparse material cultures that are prone to decay, and varying degrees of mobility that make it difficult to infer land use at a given site. Moreover, while paleo‐archives such as dung spores and fecal sterols present opportunities for reconstructing animal production, suitable sites are rare in many semiarid environments where extensive forms of animal production are common, and analyses are both time‐consuming and expensive.

## 5 A FRAMEWORK FOR CHARACTERIZING ANIMAL PRODUCTION

**Figure 2:**
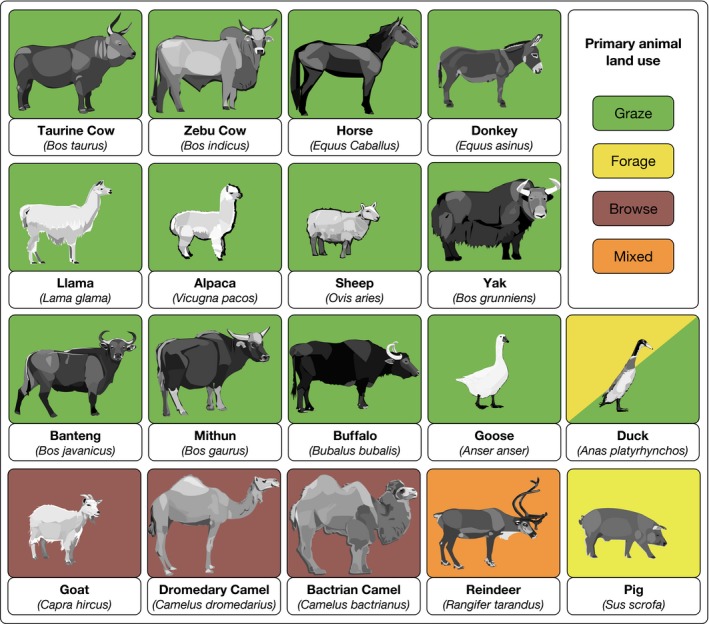
Domestic herd animals (DHA) included in animal production, and their primary land use(s). Sources include: (Aganga & Tsopito, 1998; Bayer, 1986; Blench, 2001; Bryant & Farfan, 1984; Cincotta, Van Soest, Robertson, Beall, & Goldstein, 1991; Coppock, Ellis, & Swift, 1986; Cosyns, Degezelle, Demeulenaere, & Hoffmann, 2001; Den Herder, Virtanen, & Roininen, 2004, 2008; Dereje & Udén, 2005; Gordon, 2003; Jørgensen, 2013; Lamoot, Callebaut, Demeulenaere, Vandenberghe, & Maurice, 2005a; Lamoot, Meert, & Hoffmann, 2005b; Mingongo‐Bake & Hansen, 1987; Papachristou, 1997; Papachristou, Dziba, & Provenza, 2005; Rodríguez‐Estévez, García, Peña, & Gómez, 2009; Rosenthal, Schrautzer, & Eichberg, 2012; Sanon, Kaboré‐Zoungrana, & Ledin, 2007; Serjeantson, 2009; White & Trudell, 1980). See Appendix S1 for details on distribution and livestock units [Colour figure can be viewed at wileyonlinelibrary.com]

As described above, defining and mapping land use for animal production are currently plagued by a number of deficiencies and limitations. In order to overcome these, we propose a general framework, which modifies existing work on modern livestock production systems (e.g., Otte & Chilonda, 2002; Robinson et al., 2011; Seré et al., 1996), and is suitable for application in the past, present, and future. The explicit goal of the framework is to consider and account for the animal production component of human land use; however, the structure is designed to be flexible and inclusive, in order to accommodate the mixed reality of livelihoods. Given our emphasis on addressing the neglected effects of land use on land cover, we focus on domestic herd animals (DHA), or those domesticated/tame species (Marshall, Dobney, Denham, & Capriles, 2014) that can be herded and directly fed on rangelands (Figure 2), although the framework also has the capacity to include non‐DHA species. There are three major components: (5.1) disentangling land use from land cover, (5.2) identifying animal production strategies, and (5.3) application.

### 5.1 Disentangling land cover and land use

**Figure 3:**
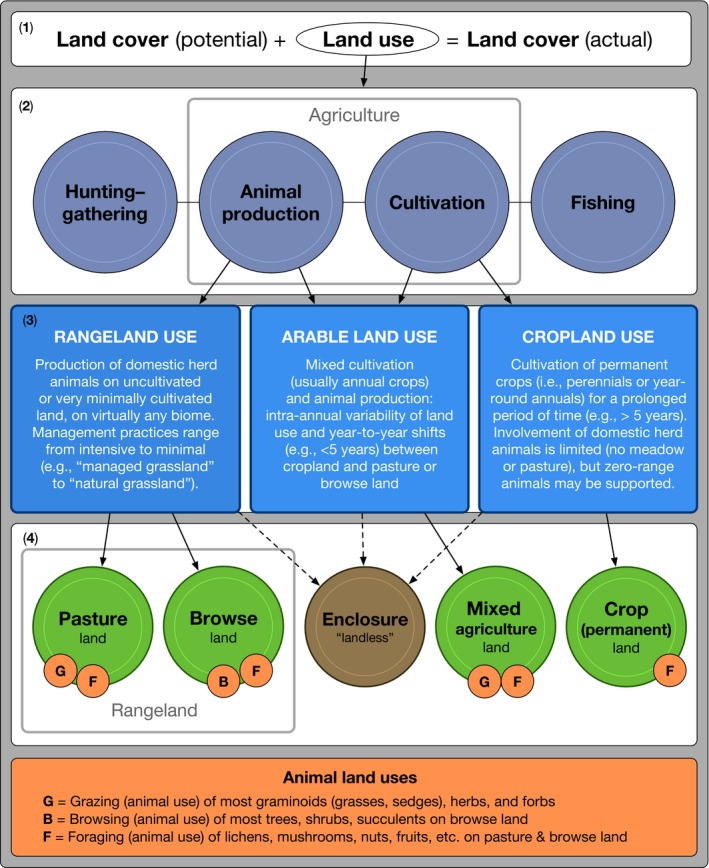
Relationships between human land use, animal land use, and land cover. See section 5.1 for a detailed explanation of this figure [Colour figure can be viewed at wileyonlinelibrary.com]

The first part of our framework aims to disentangle land use from land cover, and to explicitly define animal production in terms of land use. While a number of rangeland and pasture definitions exist (e.g., Table S1, Allen et al., 2011; FAOSTAT, 2014, Killmann, 2002), our terminology is specially defined to fit data‐driven land cover modeling applications, that is, to deal with the effects of land use for animal production on different types of land cover. This section is comprised of four subparts (1–4), depicted in Figure 3.

#### 5.1.1 Land cover and land use

**Land cover (potential)** refers to the cover that would exist without anthropogenic modification, and may include or exclude animal–plant interactions. **Land use** refers to all livelihood strategies that occur on land cover (potential) in order to produce **land cover (actual)**.

#### 5.1.2 Types of subsistence

The four main subsistence categories, which form livelihood strategies, are hunting and gathering, animal production, cultivation, and fishing. Livelihood strategies typically depend upon more than one type of subsistence, in varying relative percentages, and the boundaries between categories are not always clear. For example, in many agricultural systems, animal production and cultivation occur simultaneously (Grigg, 1974), and may be further mixed with hunting–gathering and/or fishing. These basic subsistence categories correspond to those employed by the LandUse6k initiative (Morrison, Gaillard, Madella, Whitehouse, & Hammer, 2016).

#### 5.1.3 Types of agricultural land use

The three categories of agricultural land use occur on a continuum, but must be separated into mutually exclusive categories so that they can be mapped and modeled. Category boundaries are primarily defined by the degree of reliance on animal production vs. cultivation, but also depend on the duration of consecutive cultivation, and the type(s) of land being managed (adapted from FAOSTAT, 2014, Otte & Chilonda, 2002; Seré et al., 1996). The most widely used criterion for defining “permanent” land use is five years of continuous use for a specific purpose (FAOSTAT, 2014), although we recognize that the appropriateness of the 5‐year criterion depends strongly on physical environmental constraints and livelihood context, and allow our framework to be flexible in order to fit different scenarios. Nonetheless, it is important that datasets be explicit about which criteria are used and why.

#### 5.1.4 Types of land and associated animal uses

**Figure 4:**
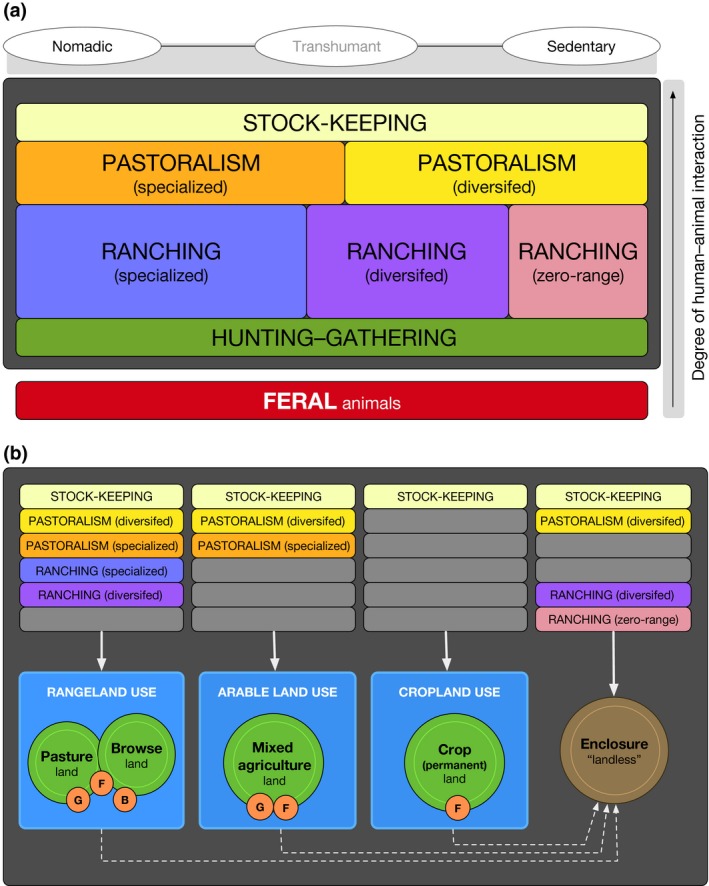
(a) Animal production exploitation strategies, and the general tendencies of mobility and human–animal interaction (note: hunting–gathering is not considered part of animal production, but is included because certain transitional systems share its features). (b) Combined consideration of exploitation strategies, human and animal land uses, and land cover. Animals may be kept in enclosures for all or part of the year, where they receive indirect feeds (dashed line) produced on other land types. For a detailed breakdown of animal land uses, see Fig. S2 [Colour figure can be viewed at wileyonlinelibrary.com]

This section of the diagram indicates the five basic types of land that can be used for animal production, and the associated animal uses that should be considered for each. **Pasture land** refers to the graminoids, herbs, and forbs that may undergo *grazing* or *foraging* animal uses by DHA as part of rangeland use; **browse land** refers to trees, shrubs, and succulents that may undergo *browsing* or *foraging* animal uses as part of rangeland use; and **mixed agriculture land** refers to land that is used for both animal production and cultivation, undergoing *foraging, grazing,* and/or *browsing* animal uses as part of arable land use. **Cropland** may undergo some *foraging* through consumption of residues (e.g., on vines or fruit trees—Foxhall, 1998), but includes negligible amounts of *grazing* or *browsing*. **Enclosure** refers to areas where animals are cooped, for example, in pens, corrals, or barns, and fed indirectly (Figure 4b). Feeds may be produced or gathered under any type of land use (Fig. S2). When referred to as a *land cover* type, **rangeland** includes all pasture land and browse land undergoing animal land uses, for the purpose of animal production. While this definition is most similar to Lambin et al. (2001), it differs in that it does not include “natural” rangelands, where only wild animals use the land. In reality, pasture land and browse land typically coexist in varying relative percentages, and should be considered this way (Verheyen, Bossuyt, Hermy, & Tack, 1999), but consideration of browse land has not been properly accounted for or disentangled from pasture in global change studies, although some datasets differentiate between shrubland and pasture (Erb et al., 2007).

### 5.2 Identification of animal production systems

Animal production consists of several different production strategies included within “ranching,” “pastoralism,” and “stock‐keeping,” which are described below. The boundaries between these strategies are not always clear, as there may be a variety of mixed and/or transitional systems present at any given time. This is why boundaries must be explicitly chosen and defined for the particular systems they describe (see Figure 4a, and Appendix S1 for discussion of transitional systems). Our classification of production systems shares some similarities with existing schemes (e.g., Otte & Chilonda, 2002; Robinson et al., 2011; Seré et al., 1996), although boundaries are based primarily on socioeconomic factors—for example, degree of animal production reliance and human–animal interaction, and the framework is explicitly designed to accommodate land use information from the past and present. Additionally, because animal production occurs under different types of human and animal land uses on virtually all types of terrestrial land cover, it is necessary to break down theses dynamic relationships and consider the explicit links between production systems and the land use and cover features discussed in section 5.1 (Figure 4b). For further consideration of animal land use, see Fig. S2.

#### 5.2.1 Ranching

Ranching systems typically involve large‐scale production of animals on enclosed and/or privately owned lands, as opposed to common property, for the primary purpose of market involvement, and are marked by human–animal relationships where people fill the role of “predator,” and animals as “prey” (Blench, 2001; Bollig & Schnegg, 2013; Ingold, 1980; Larocque, 2014; Strickon, 1965). The number of animals owned per person is usually higher than among pastoralists (e.g., Botha, 2013), and is principally limited by the size of the land or enclosure and the degree of dependence on indirect feeds (e.g., see the feed efficiencies database in Herrero et al., 2013). Ranching is the dominant animal production strategy used in North America, Australia, and some of South America (Blench, 2001; Strickon, 1965): below we distinguish three subtypes (*specialized*,* diversified*, and *zero‐range*).

##### Specialized ranching

Refers to ranching systems that depend almost entirely (e.g., 75%+) on rangelands in order to produce DHA (Figure 2), and are often associated with wild predator extirpation. These systems are characterized by the semimanagement of DHA, where animals feed directly on enclosed or private rangelands (Fig. S2), and are wrangled rather than actively managed (e.g., Rivière 1972 in Blench, 2001; Ingold, 1980; Strickon, 1965). For example, sheep and beef cattle graze and forage year‐round in much of Australia, where temperatures and low stocking densities permit this type of animal production (Wolfe, 2009), although see Appendix S1 for discussion on the transitional properties of “open range” systems.

##### Diversified ranching

Refers to ranching systems in which animals depend upon both direct and indirect feeds (i.e., >25%). Animals are often indirectly fed in enclosures for part of the year, and directly fed on enclosed rangelands for the rest of the year. Ranches such as these are common in the United States, Canada, Australia, and other high‐income countries, but encompass any system that relies on a combination of rangeland use and supplemental feeding (see Chambers, 1932; Gerth & Gerbig, 1980).

##### Zero‐range ranching

Refers to intensive ranching systems in which animals, including but not limited to those listed in Figure 2, are kept at high stocking densities in “enclosures,” and fed almost entirely with indirect feeds (similar to the “landless” category in Seré et al., 1996; Fig. S2). Examples include some commercial dairies in Europe, Australia, or North America (e.g., Annett, 2015; Wolfe, 2009), and intensive poultry or pig farms, which produce large amounts of waste (Seré et al., 1996; Sharpley, Herron, & Daniel, 2007).

#### 5.2.2 Pastoralism

Pastoralism has a wide variety of definitions. For our purposes, we define it as the *active* herding of DHA (Figure 2), marked by a relationship of animal “protection” (Ingold, 1980), and typically occurring on shared rangelands for the primary purpose(s) of subsistence, trade, and/or exchange. Herd sizes are typically limited to the number of animals that herders can manage on a given landscape, and social wealth circulation is a common feature of these systems, excepting certain transitional systems with high herd numbers (see Appendix S1). Today, pastoralism primarily occurs in Eurasia and Africa. It never developed in Australia or most of the New World, with the exception of alpaca and llama pastoralism in the Andes (Blench, 2001; Larocque, 2014; Shikui, Ruijun, Xiaopeng, & Zizhi, 2002).

##### Specialized pastoralism

Refers to livelihood systems that depend almost entirely (e.g., > 75%) on pastoralism, including goods traded/exchanged for animal products, and mobility tends toward fully mobile, or nomadism. Some Maasai and Samburu pastoralists in East Africa are archetypal examples of specialized herders (Homewood, 2008; Homewood, Kristjanson, & Trench, 2009); other highly mobile groups include Rashaida camel herders in Sudan and Al Murrah Bedouin in Saudi Arabia (see Blench 2001; Cole 1975 in Blench 2001).

##### Diversified pastoralism

Refers to mixed systems, in which livelihood strategies only partially depend on animal production (e.g., 25–75%), mobility ranges from transhumant to virtually sedentary (see sections 1.4.2 and 1.4.3 in Blench, 2001), and feeding may be direct or indirect (Fig. S2). *Diversified pastoralism* is very common, including, for example, settled or semi‐settled Fulani in Nigeria (personal observation, October, 2016), but it is particularly difficult to understand in terms of land use and cover change because it takes on so many forms, both spatially and temporally (see Figure 4a, b). For example, an archeological assemblage showing diversified pastoralism may represent one single group's approach to land use (e.g. an agro‐pastoral household), where cultivation and localized herding are both important, or it may represent several groups (agro‐pastoral, transhumant, and/or specialized) simultaneously using the land, with complex trade and exchange relations. Therefore, differentiations between *diversified pastoralism* systems need to be further explored with land use evidences, especially in the historic and prehistoric past.

#### 5.2.3 Stock‐keeping

**Figure 5:**
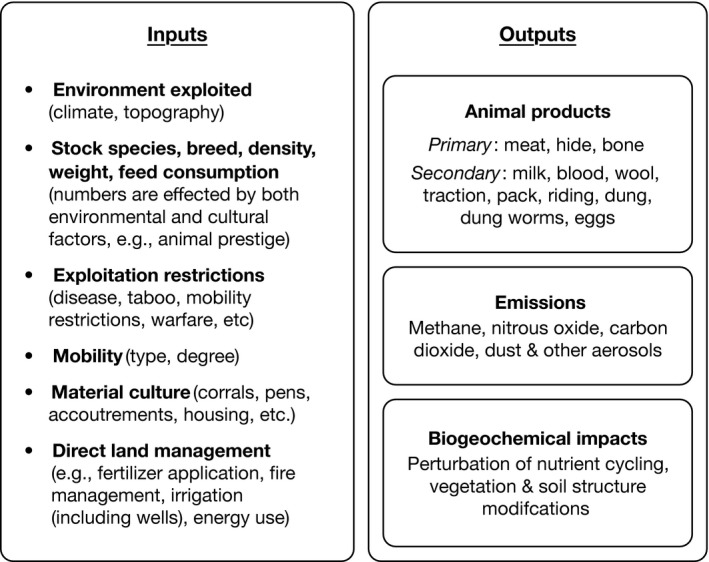
Animal production inputs and outputs that determine the intensity of land use (e.g., Blench, 2001; Dahl & Hjort, 1976; Gosden & Hather, 2005; Greenfield, 2010; Ingold, 1980)

Typically involves household production of few animals—virtually any species on any land type—for secondary products (Figure 5) and/or risk reduction strategies, that is, bet‐hedging (although see Appendix S1 for transitional systems). For example, reindeer may be stock kept as hunting decoys or for a variety of secondary purposes (Ingold, 1980). *Stock‐keeping* is also marked by a high degree of human–animal interaction, and can accompany any type of livelihood strategy, with mobility ranging from sedentary to fully mobile: for example, it is a common component of mixed crop–livestock systems today, but may also supplement hunting–gathering‐based livelihoods in the past or present (e.g., Ingold, 1980). Overall, *Stock‐keeping* is highly variable in terms of animal land use (Fig. S2).

### 5.3 Application

In order to perfectly characterize or reconstruct the land use and land cover changes associated with animal production, one would need to quantify all details of land use intensity (inputs and outputs) for a given environment (Alkemade, Reid, Van Den Berg, Leeuw, & Jeuken, 2013; Alkemade et al., 2009; De Groot, Alkemade, Braat, Hein, & Willemen, 2010; Herrero, Thornton, Gerber, & Reid, 2009; Petz et al., 2014). In reality, however, this information is not available with sufficient accuracy, and our current understanding of this complex and multidimensional process is poor: for instance, even with contemporary data, it is extremely difficult to estimate global grazing intensity (Erb et al., 2013, 2016; Kuemmerle et al., 2013). It is possible and more practical, however, to infer information about land use intensity by first identifying animal production systems, then building more detailed information about inputs and outputs (Cecchi et al., 2010; Herrero et al., 2013; Otte & Chilonda, 2002; Robinson et al., 2011; Van De Steeg, Verburg, Baltenweck, & Staal, 2010): see Figure 5 for a comprehensive list, but note that the intensity of other land uses, such as cultivation, is beyond the scope of this framework. Below, we discuss a variety of land use evidences that can be applied to this framework in order to map animal production systems in both the past and present.

#### 5.3.1 Application in the present

RS & SI data provide some information about land use, for example, especially industrial‐scale land uses like ranching; but as outlined above, they do not accurately represent all animal production. It will be important to improve the state of SI & RS data in the future—and this framework can help to ameliorate data gathering and analysis—however, there is still a clear need for incorporating other types of land use evidence in order to make more robust estimations by filling gaps or correcting SI data.

In order to distinguish land use strategies and production systems, it is necessary to estimate the degree of reliance on animal production. A useful way to determine this information in present times is with livelihoods analysis profiles: for example, Cecchi et al. (2010) used them to map production strategies in Eastern Africa. The underlying premise of livelihoods analysis is to understand the relative importance of the subsistence types that make up a livelihood, making this data gathering approach very applicable to our framework. With the Household Economy Approach (HEA), for example, production systems are grouped into three categories based on income (Seaman, Clarke, Boudreau, & Holt, 2000). For example, pastoral is ≥80% livestock, agro‐pastoral is > 50% and < 80% livestock, and mixed farming is ≤50% livestock (Cecchi et al., 2010; Robinson et al., 2011). Other advantages of livelihood analysis include the following: more data are available in GIS format, it has already been conducted for all or parts of 30 countries, it corresponds well with existing livestock production systems (Robinson et al., 2011), and if livelihoods data are not available, then other sources and expert opinion can be used (Cecchi et al., 2010; Robinson et al., 2011). For instance, detailed accounts of land use from ethnographic case studies (e.g., Dahl & Hjort, 1976) can be used to fill in production system maps, or they can be used as analogues to model land use information in places where little or no data are available. Continued livelihoods research should seek to increase spatial coverage and collect more detailed information about land use intensity where possible (Figure 5), so that this approach becomes more informative and applicable on a global scale.

It is also necessary to have a better understanding of the complex interplay between production systems and land cover change, that is, the net direction and extent of vegetation change that results from anthropogenic interventions. In order to do this, it is necessary to establish environmental baselines, (e.g. rainfall normalized NDVI values: Vågen & Gumbricht, 2012), by collecting land use and cover information in tandem. The Land Degradation Surveillance Framework (LDSF: Vågen, Winowiecki, Tamene, & Tondow, 2015; Vågen & Winowiecki, 2014)—a relatively inexpensive approach to collecting ground‐based observations—is one way to gather this type of information, and could greatly improve our understanding of the relationships between production strategies and land use intensity, that is, by comparing production system details derived from livelihoods analysis with land degradation assessments. Furthermore, this type of data collection can account for land use beneath forest canopy, which is neglected by RS data. Combined livelihoods analysis and ecosystem assessment information should also be compared with global livestock datasets (e.g., Herrero et al., 2013), in order to refine estimations and distributions of land use intensity variables, such as biomass use, feed efficiencies, and GHGs. The LDSF could be enhanced, however, by collecting more livelihoods information for cross‐validation. Overall, various sources of land use evidence should be applied to our framework, in order to improve estimations of animal production extent and intensity in the present.

#### 5.3.2 Application in the past

Disentangling the relationship between land use and land cover in prehistoric times requires the same general understanding of production systems and ecosystem health as in the present; however, the types of available data are different, the relationship between land use and cover is more ambiguous, and ranching production systems are largely irrelevant. The problem is similar for the historic past, although written records from historical maps, observations, and census data may provide valuable information, and ranching production systems become more pertinent.

In order to reconstruct animal production systems in the past, there is a need for more archeological coverage with quantifiable information, including well‐dated layers and archeological syntheses that allow characterization of animal production systems at regional to continental scales. Faunal remains (animal bones) provide one of the best evidences for animal production, and although there are some complications inherent in interpreting assemblages (Brochier, 2013; Chang & Koster, 1986), distinctions between production systems need to be explored with regional or continental databases (see, e.g., Manning et al., 2013). There is also a need for incorporating cross‐disciplinary evidence and methods in order to make reconstructions more robust, for example, combining archeological and paleoecological evidence, and/or using modern analogues to infer past land use (Biagetti, Alcaina‐Mateos, & Crema, 2016; Clarke, 2015; Dunne et al., 2012; Ejarque, Miras, & Riera, 2011; Evershed, 2008; Gaillard, Birks, Emanuelsson, & Berglund, 1992; Gifford‐Gonzalez, 1991; Graf & Chmura, 2006). For example, Conolly, Manning, Colledge, Dobney, and Shennan (2012) combined species distribution modeling with faunal and modern environmental data in order to investigate changes in the past ranges of *Bos primigenius* and *Bos taurus* in southwest Asia and Europe.

Studies of vegetation dynamics are also useful in understanding past land use (Foster, 1992; Foster et al., 2003; Verheyen, Honnay, Motzkin, Hermy, & Foster, 2003), either as a direct indicator of land use (e.g., forest biodiversity: Dupouey, Dambrine, Laffite, & Moares, 2002; Vellend, 2004), or in order to understand vegetation response to a particular type of land use, such as the effects of grazing intensity on grassland plants (Mcintyre & Lavorel, 2001; Noy‐Meir, Gutman, & Kaplan, 1989). In addition, studies on chemical soil characteristics may provide insight into past land use, and vice versa (Goodale & Aber, 2001; Verheyen et al., 1999). Thus, while inventories of specific forms of land use are highly discontinuous at global scale, drawing on evidence from a range of disciplines will facilitate more robust characterization of animal production livelihoods and landscapes.

## 6 CONCLUSION

Despite the global importance of animal production, the effects of this type of land use on land cover change have been neglected in global change studies. Estimations of global pasture area are highly variable in both the past and present, as methods are riddled with uncertainty, biases, and data quality issues (e.g., Fetzel et al., in press). While a number of production system classifications exist, they are rarely applied in this context; instead, studies of land use and cover change have been plagued by imprecise definitions and lacking characterization of production systems. Furthermore, the intensity of animal production is typically investigated in an unrealistic manner, without considering the varying effects of animal production systems on different types of land cover. In light of these deficiencies, our framework aims to clearly define animal production in terms of both land use and land cover, and to explicitly characterize animal production systems. Furthermore, our cross‐disciplinary framework has the capacity to incorporate a variety of lines of evidence for past and present land use, as well as details of land use intensity. This will allow for a more robust estimation of land use for animal production, and a more realistic consideration of its effects on land cover change in the past, present, and future.
