# Supplementary material for: Land use for animal production in global change studies: Defining and characterizing a framework
Source: Glob Chang Biol. 2017 Jun 1;23(11):4457–71. doi: 10.1111/gcb.13732 (PMC5655935; doi:10.1111/gcb.13732)
Supplement: Supplementary file 3 [file GCB-23-4457-s003.docx]

**Table S1** Details of the various land use and land cover terms used between studies. Many sources provide unclear definitions of these terms, and/or may not abide by the definition in application. Land use / land cover datasets may also rely on a variety of data sources, each with their own inherent definitions and applications. As a result, land use and land cover categories are often ambiguous.

| **Source** | **Land use term** | **Land cover term** |
| --- | --- | --- |
| (Ellis *et al.*, 2010) | pasture | rangeland |
| (Ellis & Ramankutty, 2008) | grazing, pasture | rangeland |
| (FAOSTAT, 2014) | pasture | herbaceous forage crops |
| (Klein Goldewijk *et al.*, 2010) | pasture, grazing land | grassland |
| (Klein Goldewijk, 2001) | pasture | pasture |
| (Hurtt *et al.*, 2001) | pasture, range | grassland |
| (Lambin *et al.*, 2003) | pasture | herbaceous forage crops |
| (Pongratz *et al.*, 2008) | permanent pastures | herbaceous forage crops |
| (Ramankutty *et al.*, 2008) | permanent pastures | herbaceous forage crops |
| (Steinfeld *et al.*, 2006) | permanent pastures | grasslands |
| (Lambin *et al.*, 2001) | rangelands | various land cover types |
| (Lund, 2007a, Lund, 2007b) | rangeland | rangeland |
| (Lund, 2005) | rangeland, grassland | rangeland, grassland |
| (Gregorio, 2005) | rangeland | grassland |
| (Verburg *et al.*, 2011) | grazing | rangelands |
| (Erb *et al.*, 2007) | grazing | various land cover types |
| (Klein Goldewijk & Ramankutty, 2004) | grazing land | pasture, grasslands |
| (Klein Goldewijk *et al.*, 2007) | grazing | permanent pasture, rangeland |
| (Hurtt *et al.*, 2006) | relies on HYDE | relies on HYDE |
| (Kaplan *et al.*, 2010) | relies on HYDE 3.1 | relies on HYDE 3.1 |
