# Supplementary material for: Land use for animal production in global change studies: Defining and characterizing a framework
Source: Glob Chang Biol. 2017 Jun 1;23(11):4457–71. doi: 10.1111/gcb.13732 (PMC5655935; doi:10.1111/gcb.13732)
Supplement: Supplementary file 4 [file GCB-23-4457-s004.docx]

**SUPPORTING INFORMATION: Land use for animal production in global change studies: defining and characterizing a framework**

Leanne N. Phelps ([Leanne.Phelps@unil.ch)](mailto:Leanne.Phelps@unil.ch)) & Jed O. Kaplan ([Jed.Kaplan@unil.ch)](mailto:Jed.Kaplan@unil.ch))

Institute of Earth Surface Dynamics (IDYST), University of Lausanne, Géopolis, 1015 Lausanne, Switzerland

**Table S1** Details of the various land use and land cover terms used between studies. Many sources provide unclear definitions of these terms, and/or may not abide by the definition in application. Land use / land cover datasets may also rely on a variety of data sources, each with their own inherent definitions and applications. As a result, land use and land cover categories are often ambiguous.

| **Source** | **Land use term** | **Land cover term** |
| --- | --- | --- |
| (Ellis *et al.*, 2010) | pasture | rangeland |
| (Ellis & Ramankutty, 2008) | grazing, pasture | rangeland |
| (FAOSTAT, 2014) | pasture | herbaceous forage crops |
| (Klein Goldewijk *et al.*, 2010) | pasture, grazing land | grassland |
| (Klein Goldewijk, 2001) | pasture | pasture |
| (Hurtt *et al.*, 2001) | pasture, range | grassland |
| (Lambin *et al.*, 2003) | pasture | herbaceous forage crops |
| (Pongratz *et al.*, 2008) | permanent pastures | herbaceous forage crops |
| (Ramankutty *et al.*, 2008) | permanent pastures | herbaceous forage crops |
| (Steinfeld *et al.*, 2006) | permanent pastures | grasslands |
| (Lambin *et al.*, 2001) | rangelands | various land cover types |
| (Lund, 2007a, Lund, 2007b) | rangeland | rangeland |
| (Lund, 2005) | rangeland, grassland | rangeland, grassland |
| (Gregorio, 2005) | rangeland | grassland |
| (Verburg *et al.*, 2011) | grazing | rangelands |
| (Erb *et al.*, 2007) | grazing | various land cover types |
| (Klein Goldewijk & Ramankutty, 2004) | grazing land | pasture, grasslands |
| (Klein Goldewijk *et al.*, 2007) | grazing | permanent pasture, rangeland |
| (Hurtt *et al.*, 2006) | relies on HYDE | relies on HYDE |
| (Kaplan *et al.*, 2010) | relies on HYDE 3.1 | relies on HYDE 3.1 |

**Figure S1**

**Fig. S1** The land cover category from the GLC2000 associated with the greatest fraction of pasture in  **a**) R2000 (Ramankutty *et al.*, 2008); **b**) HYDE 3.1 (Klein Goldewijk *et al.*, 2010).

**Figure S1 methods**: To establish the type of land cover upon which pasture, as defined by HYDE and R2000, is occurring, we used the GLC2000 global land cover dataset and combined this with the information in the above-mentioned land use scenarios. To achieve this, we started with the nominal 1km GLC2000 data and calculated the fraction of each of the 23 GLC2000 land cover categories on the 5 arc minute grid used by HYDE and R2000. We then developed a hierarchical classification scheme to assign the pasture fraction to the most likely GLC2000 categories. Mixed agricultural, grass-, and shrub-land were given the highest priority, forests, urban areas, and water were assigned lower priorities. The result is a series of maps showing the fraction of pasture in each of the 22 GLC2000 land cover categories, for each of HYDE and R2000. To facilitate interpretation of this multidimensional dataset, we selected the land cover category having been assigned the largest proportion of the overall pasture fraction.

Below we discuss specific differences between R2000 (Ramankutty *et al.*, 2008) and HYDE 3.1 (Kaplan *et al.*, 2010) datasets for the year 2000, in terms of pasture fraction (Fig. 1) and pasture-associated land cover type (Fig. S1):

**Saudi Arabia**: R2000 uses subnational statistics for Saudi Arabia that exclude arid lands. FAOSTAT reported 1.76 million km^2^ of permanent pasture (including arid lands), while subnational census data reported only 486 km^2^. Conversely, HYDE 3.1 includes much higher pasture fractions on “bare areas” than R200.

**China**: R2000 uses a pasture estimate for China resembling the results of Verburg and Chen (2000) totaling 2.9 million km^2^, which significantly decreases pasture area compared to the 4 million km^2^ FAO estimate. On the other hand, HYDE 3.1 uses FAO land use and subnational statistics (China National Bureau of Statistics, 2006 in Klein Goldewijk *et al.*, 2010), yielding very different land use / cover estimates between the two dataset. These differences are especially visible in Tibet, where HYDE 3.1 includes much higher percentages of pasture on “herbaceous cover” and “sparse herbaceous or sparse shrub cover”.

**India**: Both HYDE 3.1 and R2000 map an insignificant area of pasture in India, even though India is estimated to have one of the highest cattle stocking densities in the world (Robinson *et al.*, 2014). The reason for this is likely because most of India’s animal production occurs on mixed agricultural land (“cultivated and managed areas” in GLC2000), which is simply classified as cropland in both HYDE 3.1 and R2000 scenarios (Asselen & Verburg, 2012).

**Australia**: R2000 distributes permanent pasture based on satellite data and administrative sub-units, which reflect sub-national variation in land use, for example, between ranching in Queensland and aboriginal land use in Northern Territory. In contrast, HYDE 3.1 distributes pasture area by combining potential natural vegetation from the BIOME1 biogeography model (Prentice *et al.*, 1992) with weighting maps and mostly national-level administrative units, producing a map of potential vegetation estimates more than actual land use distribution. As a result, HYDE 3.1, again, includes higher pasture fractions on “bare areas”.

**Circumpolar Arctic**: very little land use for animal production, e.g., for reindeer, is accounted for by either data set: the reason for this is that both datasets largely exclude areas north of 50º.

**Western North America**: R2000 disperses pasture area across the western United States, while HYDE 3.1 estimates much lower pasture fractions in the Rocky Mountain area, Nevada and Utah, and concentrates it eastwardly in the Plains, Southwest and northern Mexico. This likely reflects similar methodological differences as discussed for Australia.

**Figure 2** (supporting information):

**Modern distributions of domestic herd animals** (DHA – Fig. 2): Taurine cattle, sheep, goat, horse, donkey, pig, goose, and duck may all be found worldwide today (for traditional pastoral ranges see (Blench, 2001). *Bos*: **Zebu cattle** are traditionally present in Africa and central and southern Asia; the **Banteng** is found primarily in southeast Asia; **Mithun** are found primarily in south and southeast Asia; and **Yak** are traditionally found in Highland Central Asia but may also be found in Europe in modern times. *Camelidae*: **Dromedary camels** are traditionally found in Africa and the middle east, but are also found in south Asia and Australia (e.g. Bell, 2013), while **Bactrian camels** are traditionally found in East-central Asia, they may also be found middle east in modern times; **llama** and **alpaca** are traditionally found in the Andes, and more recently in Europe and North America. **Buffalo** are found throughout Eurasia and Australia in recent times. **Reindeer** are found primarily in circumpolar Eurasia, and more recently in North America (Blench, 2001 - Table 2, GBIF, 2017). See Larson & Fuller (Larson & Fuller, 2014) for animal domestication information, however in order to specify historic and prehistoric distributions, it is necessary to assemble global faunal databases.

**Livestock units**: many modern studies represent animal weight with standardized livestock units (LUs), e.g., where one (OECD-member country) cow is equivalent to eight (OECD) sheep or goat (0.125 LUs each), but a sub-Saharan cow equals 0.46 LUs, or a Central/South American cow equals 0.75 LUs (Herrero *et al.*, 2013, Seré *et al.*, 1996). This method is useful for a given point in time, where accurate global livestock weight averages are available. When modeling animal production over long periods of time, however, it may be useful to use actual weights (or weight ranges), which can respond to environmental factors (e.g. Illius & O’Connor, 2000, Pachzelt *et al.*, 2013). Moreover, the feed efficiencies and vegetation selection tendencies of different animal species should also ideally be incorporated into modeling scenarios, i.e. by considering actual feed intake, vegetation digestibility, breeding and mortality, etc. (Illius & O’Connor, 2000, Pachzelt *et al.*, 2013). Only primary animal land uses are noted in figure 2, however vegetation selection varies between species and depends upon availability, and this variability should ideally be accounted for: see the sources cited in figure 2 for a number of examples.

**Transitional systems** (section B supporting information):

Animal production occurs on a continuum with some systems falling between two production strategies. Boundaries for these must be explicitly chosen: some examples are given below:

*Pastoral to ranching:* these systems have features of both pastoralism and ranching. For example, modern animal production systems in the European Alps are often enclosed within fence boundaries (Blench, 2001) similar to ranching, but are driven by transhumant mobility strategies and may have closer human management, which is more similar to pastoralism. Moreover, enclosed ranching systems may use rotation and micro-mobility techniques that function similarly to pastoralism (LaRocque, 2014).

*Pastoral to hunting:* these systems have features of both pastoralism and hunting. Prime examples include the large reindeer herds of the Sel’kups, Ostyak, Voguls and Skolt Lapps, which are herded in summer, but may roam free after fawning and are only hobbled during winter to prevent long distance travel (Ingold, 1980). Furthermore, these “pastoral” systems tend to lack wealth circulation strategies typical of pastoralism.

*Ranching to hunting (and feral*): an example of a system that includes transitional features of both ranching and hunting is open range cattle ranching in North America that was common around the nineteenth century. This type of system involves free-ranging prey and “cow hunts” on communal lands (Coughenour, 1991, Ingold, 1980, LaRocque, 2014), which are indicative of hunting, but instead of wild animals, the prey consist of minimally managed DHA, similar to ranching, but production is of relatively little importance and oriented to local markets (Strickon, 1965). These systems typically develop out of pastoral practices; however, land use patterns more closely reflect a cross between hunting and ranching than pastoralism and ranching, which is why we classify it this way. Another example comes from the Rio Branco region of northern Brazil, where herds of cattle filled a vacant niche and populated the land: some became feral, while others were regularly rounded up for subsistence purposes (Rivière, 1972 in Blench, 2001, Ingold, 1980). The effects of feral animals on land cover must also be considered (e.g. pigs in Nogueira-Filho *et al.*, 2009).

*Ranching to Stock keeping*: in East Africa, there are intensive, yet small-scale dairy farms (described as non-traditional “smallholder dairy systems” in Otte & Chilonda, 2002): like enclosed, zero-range stock keeping systems, producers keep few stall-fed cattle, typically under ten (Orodho, 2005). Yet, like zero-range ranching, however, these systems are important and common parts of the regional economy of East Africa.

**Figure S2**:

Four different types of feed are considered in Herrero et al (2013): 1) grain, 2) grass: direct grazing or as silage, 3) occasional feeds, e.g. cut-and-carry forages and legumes, roadside grasses, 4) fibrous crop residues. These distinctions are very important for determining feed efficiencies, but in order to accurately account for the effects of land use on cover, it is also necessary to separate direct and indirect animal land uses. The production of indirect feeds varies significantly: therefore, in order to account for the effects of these “open” systems (Seré *et al.*, 1996) on the environment, it is necessary to understand feed composition and the land use from which feeds are produced: although we consider these relationships (fig. S2), this framework does not account for the effects of land uses other than animal production.

**Rangeland**: *direct feeding* on rangelands involves the grazing, browsing and/or foraging of pasture-land (graminoids, herbs, forbs) and browse-land (trees, shrubs, and succulents) by DHA. *Indirect feeding* occurs when feeds are gathered or mowed by humans, and fed to tethered or cooped animals, e.g. including cut-and-carry forages or legumes, hay, and silage.

**Mixed agriculture land**: *direct feeding* on mixed agriculture land involves a variety of animal land uses, including grazing or foraging of graminoids, herbs and forbs by DHA, and the foraging of crop products, e.g. residues / stovers by any domestic/tame animal. *Indirect feeding* occurs when crops or residues, e.g. grains or stovers, are cultivated and fed to animals; it may also occur where graminoids, herbs, and forbs are gathered / mowed and fed to tethered or cooped animals.

**Cropland** (permanent): d*irect feeding* on cropland is relatively rare, but involves foraging permanent crops, for example, vines or fruit trees (Foxhall, 1998). *Indirect feeding* on cropland is much more common, and includes any crop products or residues that are fed to animals.

**Enclosure**: all animal feeds are indirect where animals are kept in enclosures, and must be cultivated on other land types.

**Fig. S2** Possible relationships between the mode of animal feeding (direct and indirect), the type of animal land use (G = grazing, B = browsing, F = foraging), animal production strategy, and land type. Note: this figure does not consider crop/animal raiding, predation or destruction, e.g., from feral and wild animals or pastoralist-farmer conflict, though these can significantly reduce production.

**References**

Asselen SV, Verburg PH (2012). A land system representation for global assessments and land-use modeling. *Global Change Biology,* **18**, 3125-3148.

Bell S (2013). Australia, home to the world’s largest camel herd. In: *BBC News*. Available at: http://www.bbc.com/news/magazine-22522695 (accessed February 2017).

Blench R (2001). *'You can't go home again': pastoralism in the new millennium*, Overseas Development Institute, London, UK.

Coughenour MB (1991). Spatial components of plant-herbivore interactions in pastoral, ranching, and native ungulate ecosystems. *Journal of Range Management,* **44**, 530-542.

Ellis EC, Klein Goldewijk K, Siebert S, Lightman D, Ramankutty N (2010). Anthropogenic transformation of the biomes, 1700 to 2000. *Global Ecology and Biogeography,* **19**, 589-606.

Ellis EC, Ramankutty N (2008). Putting people in the map: anthropogenic biomes of the world. *Frontiers in Ecology and the Environment,* **6**, 439-447.

Erb K-H, Gaube V, Krausmann F, Plutzar C, Bondeau A, Haberl H (2007). A comprehensive global 5 min resolution land-use data set for the year 2000 consistent with national census data. *Journal of Land Use Science,* **2**, 191-224.

FAOSTAT (2014). Glossary list. Available at: <http://faostat.fao.org/site/375/default.aspx> (accessed November 2016), Food and Agriculture Organization of the United Nations.

Foxhall L (1998). Snapping up the unconsidered trifles: the use of agricultural residues in ancient Greek and Roman farming. *Environmental Archaeology,* **1**, 35-40.

GBIF (2017). GBIF backbone taxonomy. Available at: <http://www.gbif.org/species> (accessed February 2017), GBIF Secretariat.

Gregorio AD (2005). *Land cover classification system: classification concepts and user manual software version (2), pp. 190.* Food and Agriculture Organization of the United Nations, Rome.

Herrero M, Havlík P, Valin H *et al.* (2013). Biomass use, production, feed efficiencies, and greenhouse gas emissions from global livestock systems. *Proceedings of the National Academy of Sciences,* **110**, 20888-20893.

Hurtt GC, Frolking S, Fearon MG *et al.* (2006). The underpinnings of land-use history: three centuries of global gridded land-use transitions, wood-harvest activity, and resulting secondary lands. *Global Change Biology,* **12**, 1208-1229.

Hurtt GC, Rosentrater L, Frolking S, Moore B (2001). Linking remote-sensing estimates of land cover and census statistics on land use to produce maps of land use of the conterminous United States. *Global Biogeochemical Cycles,* **15**, 673-685.

Illius AW, O’connor TG (2000). Resource heterogeneity and ungulate population dynamics. *Nordic Society Oikos,* **89**, 283-294.

Ingold T (1980). *Hunters, pastoralists and ranchers: reindeer economies and their transformations,* 326 pp. Cambridge University Press, Cambridge.

Kaplan JO, Lemmen C, Klein Goldewijk K *et al.* (2010). Holocene carbon emissions as a result of anthropogenic land cover change. *The Holocene,* **21**, 775-791.

Klein Goldewijk K (2001). Estimating global land use change over the past 300 years: the HYDE database. *Global Biogeochemical Cycles,* **15**, 417-433.

Klein Goldewijk K, Beusen A, Janssen P (2010). Long-term dynamic modeling of global population and built-up area in a spatially explicit way: HYDE 3.1. *The Holocene,* **20**, 1-9.

Klein Goldewijk K, Ramankutty N (2004). Land cover change over the last three centuries due to human activities: the availability of new global data sets. *GeoJournal,* **61**, 335-344.

Klein Goldewijk K, Van Drecht G, Bouwman AF (2007). Mapping contemporary global cropland and grassland distributions on a 5 × 5 minute resolution. *Journal of Land Use Science,* **2**, 167-190.

Lambin EF, Geist HJ, Lepers E (2003). Dynamics of land-use and land-cover change in tropical regions. *Annual Review of Environment and Resources,* **28**, 205-241.

Lambin EF, Turner BL, Geist HJ *et al.* (2001). The causes of land-use and land-cover change: moving beyond the myths. *Global Environmental Change,* **11**, 261-269.

Larocque O (2014). Revisiting distinctions between ranching and pastoralism: a matter of interspecies relations between livestock, people, and predators. *Critique of Anthropology,* **34**, 73-93.

Larson G, Fuller DQ (2014). The evolution of animal domestication. The Annual Review of *Ecology, Evolution, and Systematics,* **45**, 115-136.

Lund HG (2004). Considerations for developing U.S. standard definitions of forest and rangeland. Report prepared for Meridian Institute, Contract No. 0045-A. Project No. 9147.8, Forest Information Services, Rev. Gainesville, VA, 108 pp. Available at: http://fhm-server.lv-hrc.nevada.edu//fia/ab/issues/Final_report_F-R_7May05. doc.

Lund HG (2007a). Accounting for the world's rangelands. *Rangelands,* **29**, 3019.

Lund HG (2007b). Separating the cows from the trees: toward development of national definitions of forest and rangeland. In: *2005 Proceedings of the Seventh Annual Forest Inventory and Analysis Symposium.* (eds Mcroberts RE, Reams GA, Van Deusen PC, Mcwilliams WH), pp. 11-20. United States Department of Agriculture, Forest Service, Portland, ME.

Nogueira-Filho SLG, Nogueira SSC, Fragoso JMV (2009). Ecological impacts of feral pigs in the Hawaiian islands. *Biodiversity Conservation,* **18**.

Orodho AB (2005). Intensive forage production for smallholder dairying in East Africa. In: *Grasslands: developments, opportunities, perspectives.* (eds Reynolds SG, Frame J), pp. 433-460. Science Publishers, Inc, Enfield, USA.

Otte MJ, Chilonda P (2002). Cattle and small ruminant production systems in sub-Saharan Africa: a systematic review. In: *Food and Agricultre Organization of the United Nations,* 99 pp. Food and Agriculture Organization of the United Nations, Rome.

Pachzelt A, Rammig A, Higgins S, Hickler T (2013). Coupling a physiological grazer population model with a generalized model for vegetation dynamics. *Ecological Modelling,* **263**, 92-102.

Pongratz J, Reick C, Raddatz T, Claussen M (2008). A reconstruction of global agricultural areas and land cover for the last millennium. *Global Biogeochemical Cycles,* **22**, GB3018.

Prentice CI, Cramer W, Harrison SP, Leemans R, Monserud RA, Solomon AM (1992). Special paper: a global biome model based on plant physiology and dominance, soil properties and climate. *Journal of Biogeography,* **19**, 117-134.

Ramankutty N, Evan AT, Monfreda C, Foley JA (2008). Farming the planet: 1. geographic distribution of global agricultural lands in the year 2000. *Global Biogeochemical Cycles,* **22**, GB1003.

Robinson T, Wint W, Conchedda G *et al.* (2014). Mapping the global distribution of livestock. *PLoS ONE,* **9**, e96084.

Seré C, Steinfeld H, Groenewold J (1996). *World livestock production systems: current status, issues and trends*, Food and Agriculture Organization of the United Nations.

Steinfeld H, Gerber P, Wassenaar T, Castel V, Rosales M, De Haan C (2006). *Livestock's long shadow: environmental issues and options*, Food and Africulture Organization of the United Nations*,* Rome.

Strickon A (1965). The Euro-American ranching complex. In: *Man, culture, and animals: the role of animals in human ecological adjustments.* (eds Leeds A, Vayda AP), pp. 229-258. American Association for the Advancement of Science, Washington, D.C.

Verburg PH, Chen Y (2000). Multiscale characterization of land-use patterns in China. *Ecosystems,* **3**, 369-385.

Verburg PH, Neumann K, Nol L (2011). Challenges in using land use and land cover data for global change studies. *Global Change Biology,* **17**, 974-989.
